# Altered Effective Connectivity of Resting-State Networks by Tai Chi Chuan in Chronic Fatigue Syndrome Patients: A Multivariate Granger Causality Study

**DOI:** 10.3389/fneur.2022.858833

**Published:** 2022-06-03

**Authors:** Yuanyuan Li, Kang Wu, Xiaojie Hu, Tianjiao Xu, Zongheng Li, Yong Zhang, Kuangshi Li

**Affiliations:** ^1^Department of Neurology and Stroke Center, Dongzhimen Hospital, Beijing University of Chinese Medicine, Beijing, China; ^2^Department of Rehabilitation, Dongzhimen Hospital, Beijing University of Chinese Medicine, Beijing, China

**Keywords:** Tai Chi Chuan, chronic fatigue syndrome, fMRI, resting-state networks, Granger causality analysis

## Abstract

Numerous evidence has shown that patients with chronic fatigue syndrome (CFS) have changes in resting brain functional connectivity, but there is no study on the brain network effect of Tai Chi Chuan intervention in CFS. To explore the influence of Tai Chi Chuan exercise on the causal relationship between brain functional networks in patients with CFS, 21 patients with CFS and 19 healthy controls were recruited for resting-state functional magnetic resonance imaging (rs-fMRI) scanning and 36-item Short-Form Health Survey (SF-36) scale assessment before and after 1month-long training in Tai Chi Chuan. We extracted the resting brain networks using the independent component analysis (ICA) method, analyzed the changes of FC in these networks, conducted Granger causality analysis (GCA) on it, and analyzed the correlation between the difference causality value and the SF-36 scale. Compared to the healthy control group, the SF-36 scale scores of patients with CFS were lower at baseline. Meanwhile, the causal relationship between sensorimotor network (SMN) and default mode network (DMN) was weakened. The above abnormalities could be improved by Tai Chi Chuan training for 1 month. In addition, the correlation analyses showed that the causal relationship between SMN and DMN was positively correlated with the scores of Role Physical (RP) and Bodily Pain (BP) in CFS patients, and the change of causal relationship between SMN and DMN before and after training was positively correlated with the change of BP score. The findings suggest that Tai Chi Chuan is helpful to improve the quality of life for patients with CFS. The change of Granger causality between SMN and DMN may be a readout parameter of CFS. Tai Chi Chuan may promote the functional plasticity of brain networks in patients with CFS by regulating the information transmission between them.

## Introduction

Chronic fatigue syndrome (CFS) is a complex disease with a 0.2 to 2.6% prevalence rate in modern society (1). Patients with CFS usually suffer from continuous fatigue. Besides, the disease is often accompanied by sleep disorder, physical pain, exercise intolerance, cognitive dysfunction, anxiety, and other symptoms, with a consequence of serious physical and mental damage. The study by Schweitzer et al. (2) shows that CFS seriously affects the patients' quality of life, and the social model of disability of CFS patients is comparable to that of the group of stroke patients and advanced cancer patients. Until now, the etiology and pathogenesis of CFS are still unclear, and most researchers identify it today as the result of a multisystem dysfunction (3–5).

In recent years, with the proposal of central sensitization mechanism and the discovery of central nervous system's injury symptoms in CFS (6–10), it has become a new research highlight to explore the changes of brain function in patients by using functional magnetic resonance imaging (fMRI). It has been more recognized that brain dysfunction is one cause of CFS symptoms, especially the abnormality of intrinsic functional connectivity (FC) in resting-state networks (RSNs). These RSNs are composed of structurally separated but functionally connected brain regions (11), which not only play a central role in normal brain functions but also in some brain diseases (12). There are several RSNs observed in the past decades, including default mode network (DMN), sensorimotor network (SMN), left frontoparietal network (LFPN), right frontoparietal network (RFPN), executive control network (ECN), visual network (VN), auditory network (AN), salience network (SN), cerebellum network, and language network, continuously sharing information with each other and associated with the processing of cognition, emotion, action, and so on (13). A study based on DMN showed that DMN in CFS patients was impaired, which was characterized by irregular posterior cingulate cortex (PCC) activity and weakened FC of bilateral inferior parietal lobules (14). Another study (15) demonstrated that, compared to healthy women, FC between DMN and frontal lobe in CFS women decreased, while FC between SN, left temporal lobe area and medulla oblongata increased. Furthermore, the findings of a previous study (16) indicated that the FC of several brain networks in female patients with CFS was impaired, including LFPN, SMN, and SN, among which the impairment of SN showed a decline in FC with PCC. PCC is one of the important nodes of DMN. In brief, while there are a few CFS-related fMRI studies, the results, especially whether the FC of DMN is damaged or not, are still under debate. Moreover, most research focused on the intrinsic FC of a single network and neglected the interaction of extensive RSNs.

In the treatment of CFS, exercise therapy is applied as the most common measure at present (17–19). Nevertheless, given its high cost and unbearable intensity caused by the symptoms such as fatigue and pain, conventional exercise therapy is unavailable for access to clinics (19, 20). New approaches are needed for helping patients to embrace to reduce chronic fatigue and pain, improving their physical and psychological function and quality of life. As an ancient discipline involving exercise, Tai Chi Chuan is a kind of complex, multicomponent mind-body intervention with low and medium intensity, which takes advantage of safety, low cost, and is widely applicable (21), as well as provides therapeutic benefits involving improving quality of life, physical function, pain management, balance and risk of falls reduction, enhancing immune response, and improving flexibility, strength, and kinesthetic sense (22). In addition, it is reported that the Tai Chi Chuan group had a higher continuance rate at the end of the intervention and a lower drop-out rate for the ongoing class than the conventional exercise group (23). A meta-analysis (24) has also proved that Tai Chi Chuan is more beneficial in relieving fatigue than conventional treatment and low-impact exercise control. The symptoms mentioned earlier that can be alleviated by Tai Ji Chuan such as fatigue, pain, dyskinesia, and so on, are related to CFS. Other encouraging evidence (25, 26) has revealed that Tai Chi Chuan can alleviate fibromyalgia (FM) more effectively than aerobic exercise, whose symptoms are extremely similar to CFS. Some researchers believe that FM and CFS share the same pathophysiological mechanism (27–29). Thus, in our hypothesis, it might also contribute to relieving the symptoms of CFS and become an effective alternative therapy for CFS.

Positively, what can support our hypothesis are the research with fMRI revealing that Tai Chi Chuan promotes the plasticity of brain function. Brain plasticity refers to the ability to change brain structure and function under the influence of the environment (30). More evidence (31, 32) confirmed that Tai Chi Chuan had a stronger ability to remodel brain function than general aerobic exercise, which was mainly reflected in the enhancement of FC between the left middle frontal gyrus and left parietal lobe. By comparing the effects of long-term Tai Chi Chuan training and long-term walking training on the brain networks of elderly women, another work (33) found that these two kinds of exercise modes could enhance the FC of DMN, SMN, and visual network (VN) with different promotion forms. Therefore, Tai Chi Chuan has some effect on improving brain function, which requires more longitudinal studies to prove.

As mentioned earlier, there are many abnormal intrinsic FCs of RSNs in CFS patients, but there is no research studying the interaction between them. A study about Tai Chi Chuan intervention in CFS is needed accordingly. Furthermore, to move beyond the identification of regional activations toward the characterization of functional circuits is a key challenge in neuroscience. Therefore, Granger causality analysis (GCA), as a powerful method to achieve this, can not only evaluate the FC of brain networks but also examine abnormal relationships among RSNs in psychiatric patients to better understand the neurobiological basis of the disorders (34–37). In this study, we will take advantage of fMRI and GCA to study the interaction between CFS patients and healthy human brain networks. By introducing Tai Chi Chuan as an effective therapy, we would expect an improvement of the symptoms in CFS patients and its promotion of the plasticity in RSNs.

## Materials and Methods

### Participants

In this study, 21 CFS patients (experimental group, 6 males and 15 females, average age 37.47 ± 12.14) and 19 healthy subjects (healthy control group, 7 males and 12 females, average age 33.31 ± 12.46) were recruited from Dongzhimen Hospital, Beijing University of Chinese Medicine. They were all right-handed, aged 25 to 65, with no previous practice history of Tai Chi Chuan and no difficulty answering and filling out the questionnaire. They had not taken psychotropic drugs for nearly a month, with no metal in the body and no contraindications for MRI examination. The experimental group met the following inclusion criteria: diagnosed as CFS of the 1994 Center for Disease Control and Prevention Case Definition (38); the course of CFS for more than 6 months; and with chronic fatigue that cannot be explained by other current diseases or drug side effects. We excluded those with major diseases, current or past psychiatric disorders, severe obesity (body mass index > 45), and any brain structure damage or abnormalities identified by MRI examinations. What's more, patients who had taken vasodilators in the past 2 weeks, who had participated in similar neuroimaging experiments within 1 month, and women who were pregnant, lactating, or menstruating were also excluded. Signed informed consent was obtained from all participants. This study was approved by the Medical Ethics Committee of Dongzhimen Hospital of Beijing University of Chinese Medicine (DZMEC-KY-2019-195).

### Health-Related Quality of Life Evaluation

The 36-item Short-Form Health Survey (SF-36) was used to evaluate the health-related quality of life (HRQoL) of all subjects, which was regarded as the main index. All subjects made a self-report with an SF-36 scale before and after training in Tai Chi Chuan. This study adopted the Chinese version translated in 1991 by the Department of Social Medicine, Zhejiang University Medical College. The scale consists of eight subscales, namely Physical Functioning (PF), Role Physical (RP), Bodily Pain (BP), General Health (GH), Vitality (VT), Social Functioning (SF), Role Emotional (RE), and Mental Health (MH). The score ranges from 0 to 100. The higher the score, the better the HRQoL.

### MRI Scanning

Before and after training in Tai Chi Chuan, all the subjects underwent an MRI scanning in Dongzhimen Hospital of Beijing University of Chinese Medicine with a MAGNETOM Prisma magnetic resonance scanner (Siemens, Germany). Before the scan, the patients were told in detail about the scanning time, scanning purpose, and precautions. After getting used to the indoor environment of MRI, they were told to rest on their backs for 30 min and wait until the participants were completely calm before starting scanning. Safety indicators (blood pressure, respiratory rate, heart rate, etc.) were monitored before and after each scan to evaluate the safety of the test process.

The two MRI scans adopted the same scanning sequence. The participants lay flat for 30 min to maintain complete calm. Then, they were required to stay still, think of nothing, keep eyes closed and refrain from falling asleep during scanning. In the process, earplugs were worn for noise isolation and the foam head holders were immobilized to minimize head movements. After that, the T1 structure image which is for 4 min and 10 s and the DTI image which is for 5 min and 10 s were continued.

Scanning parameters: fMRI was applied with echo-planar imaging (EPI) sequence: Repetition time (TR), 2000 ms; Echo time (TE), 30 ms; Matrix, 64 × 64; Field of view (Fov), 225 × 225 mm; Slice thickness, 3.5 mm; Gap, 0.7 mm; Phase encode direction, A>>P; Flip Angle, 90°; Fat suppr, Fat sat. The three-dimensional structure imaging scan of the whole brain was scanned using T1W1 sequence: TR, 1900 ms; TE, 2.53 ms; Fov, 250 × 250 mm; Matrix, 256 × 256; Slice thickness, 1.0 mm.

### Tai Chi Chuan Training Program

To eliminate the influence of different training durations and frequencies, the training time of Tai Chi Chuan in the experimental group and the healthy control group were designed in the same way. Both the experimental group and the healthy control group were given Tai Chi Chuan training twice a week for 1 h each time. The whole process was under the guidance of therapists, with the exercise learning and repeated posture control training. The course of treatment was 4 weeks, with eight training courses in total. For the rest of the time, each participant was asked to practice Tai Chi Chuan for 30 min every day. Training Tai Chi Chuan standard movements referred to the 24-style simplified version of the General Administration of Sport of China and the Tai Chi Chuan's standard movements in Chinese Traditional Health Care Sports and Health Preservation, national teaching materials for colleges and universities citation. We selected three experienced Tai Chi Chuan coaches for systematic training, and let them know the intervention methods of Tai Chi Chuan and the basic knowledge of related diseases. During the experiment, Tai Chi Chuan's teaching was recorded to ensure the practice quality. After each training, the participants were reminded to carry out family Tai Chi Chuan exercises, video feedback of each exercise, and telephone follow-up supervision training during the research process.

### Data Processing

All data processing was completed by DPABI (http://rfmri.org/dpabi) (39).

#### Anatomical Data Preprocessing

The T1 images were converted into the BIDS dataset. Then, they were corrected for intensity non-uniformity with N4BiasFieldCorrection (40), which was provided by Advanced Normalization Tools (ANTs) 2.3.3. The derived images were skull-stripped with OASIS30ANTs as the target template. The remaining brain tissues were segmented into the cerebrospinal fluid (CSF), white matter (WM), and gray matter (GM) by the BET (FSL 5.0.9). Brain surfaces were reconstructed using recon-all (FreeSurfer 6.0.1). A classic method, which reconciles ANTs-derived and FreeSurfer-derived segmentation of the cortical gray matter of Mindboggle (41), was applied for refining the brain mask estimated previously. Volume-based spatial normalization to one standard space (Montreal Neurological Institute, MNI) was performed through nonlinear registration with antsRegistration (ANTs 2.3.3), using brain-extracted versions of both T1 reference and the T1 template. Meanwhile, ICBM 152 Nonlinear Asymmetrical template version 2009c was selected for spatial normalization.

#### Functional Data Preprocessing

First, the custom methodology of fMRIPrep (42) was used to generate the reference volume and its skull-stripped version. Susceptibility distortion correction (SDC) was omitted. Bbregister (FreeSurfer), which implements boundary-based registration, was applied for co-registering the fMRI reference and T1 reference. Moreover, slice-time was corrected using 3dTshift from AFNI and spatiotemporal filtering was conducted by mcflirt (FSL). The BOLD time series were resampled into standard space and generated a preprocessed BOLD run in MNI space. At the same time, framewise displacement (FD), DVARS, and three region-wise global signals were calculated by the preprocessed BOLD. In addition, a set of physiological regressors were extracted to allow for the component-based noise correction (CompCor). Above components were dropped from the BOLD, and frames that exceeded a threshold of 0.5 mm FD or 1.5 standardized DVARS were annotated as motion outliers. Gridded (volumetric) resampling was performed using antsApplyTransforms (ANTs), configured with Lanczos interpolation to minimize the smoothing effects of other kernels. Non-gridded (surface) resampling was performed using mri_vol2surf (FreeSurfer).

### RSNs Extraction

Independent component analysis was applied to extract the RSNs by GIFT software (University of New Mexico, Albuquerque, NM). The number of independent components in all data was calculated by the method of the minimum description length (MDL) technique. Randlnit and Bootstrap operations were applied to evaluate the independent components. Then, we selected the brain networks by combining the manual selection, goodness-of-fit method (43), and the evidence which was observed in previous literature. Meanwhile, the relationship between CFS symptoms and brain network functions was also a factor to be considered. We selected six specific networks including executive control network (ECN), visual network (VN), sensory motor network (SMN), right frontoparietal network (RFPN), default mode network (DMN), and salience network (SN).

### Network Analysis

To analyze the changes of FC in these six brain networks before and after Tai Chi Chuan, the images of components were normalized to Z-scores with Fisher's r-to-z transformation for acquiring the entire brain Z-score map of each subject. Meanwhile, a repeated measures model, including condition effect and interaction effect, was used to investigate the Tai Chi Chuan-induced changes in the six brain networks. The significant thresholds were set as 0.005 and family-wise error (FWE) correction for multiple comparisons at *p* = 0.05 at the cluster level was applied.

### Network Granger Analysis

All the selected components were filtered between 0.01 and 0.1 Hz for multivariate Granger causal modal to explore the characteristics of networks. Meanwhile, we used the generalized partial directed coherence (GPDC) as the measured parameter (44). The method of the Akaike information criterion was applied for determining the order of Granger causality analysis. Then, comparisons between groups were done on the causal interaction of six components. We also conducted a one-sample *t*-test in each component to compute the single network imaging. An independent *t*-test was used for comparison between groups and a paired *t*-test was used for intragroup comparison. The *P*-value of the *t*-test was set as 0.05 which was corrected by the false discovery rate (FDR) for multiple comparisons. Finally, BrainNetViewer was used to display the result onto a 3D brain surface.

### Correlation Analysis

Compared to the healthy subjects, there was a significant decrease in the causal relationship between SMN and DMN in CFS patients. However, after 1 month-long training in Tai Chi Chuan, the causal relationship between SMN and DMN in CFS patients was enhanced. Given the relevance of SMN and DMN to sensorimotor function, which could be best reflected by RP and BP scores, we conducted a correlation analysis between the mean Granger causality value of SMN-DMN and RP and BP scores in CFS patients. Statistical analyses were conducted using SPSS 20.0, and the threshold was set at *P* < 0.05.

## Results

### Demographic and Clinical Information

In this study, 21 CFS patients (experimental group) and 19 healthy people (healthy control group) were recruited from Dongzhimen Hospital of Beijing University of Chinese Medicine. There were 6 males and 15 females in the experimental group, with an average age of 37.47 ± 12.14 years and an average body mass index (BMI) of 21.88 ± 3.49. The healthy control group consisted of 7 males and 12 females, with an average age of 33.31 ± 12.46 years and an average BMI of 22.98 ± 2.83. There is no statistical difference in sex, age, and BMI between the two groups. Refer to Table 1 for details.

**Table 1:** Comparison of sex, age, and BMI between the experimental group and the healthy control group.

**Group**	**Experimental group** **(*N* = 21)**	**Healthy control group** **(*N* = 19)**	***P-*value**
Male/Female	6/15	7/12	0.557
Age ($\bar{x}$ *± s*)	37.47 ± 12.14	33.31 ± 12.46	0.29
BMI ($\bar{x}$ *± s*)	21.88 ± 3.49	22.98 ± 2.83	0.75

By observing the scores on SF-36 scale, we found that at baseline, the scores of PF, RP, BP, GH, VT, SF, and RE in the experimental group were significantly lower than those in the healthy control group (*P* < 0.05), while there was no significant difference in the scores of MH between the two groups (*P* > 0.05). After 1month-long training in Tai Chi Chuan, the scores of PF, RP, BP, GH, VT, MH, SF, and RE in SF-36 in the experimental group were significantly higher than those before the training (*P* < 0.05). There was no obvious change in the SF-36 scale score of the healthy control group after practicing Tai Chi Chuan (*P* > 0.05). Refer to Table 2 for details.

**Table 2:** Comparison of SF-36 scores between the experimental group and the healthy control group before and after Tai Chi Chuan exercise.

**SF-36**	**Experimental group** **(*****N*** **=** **21)** **(Mean** **±SD)**		**Healthy control group** **(*****N*** **=** **19)** **(Mean** **±SD)**	
	**Before**	**After**	**Before**	**After**
MH	64.38 ± 16.39	73.52 ± 13.71^#^	72.63 ± 16.24	75.36 ± 16.18
VT	58.09 ± 16.99*	75.00 ± 15.08^#^	74.21 ± 14.83	77.89 ± 16.69
PF	85.47 ± 12.64^*▴^	93.33 ± 8.42^#▴^	95.79 ± 3.82	97.11 ± 7.13
RP	30.95 ± 37.00^*▴^	83.33 ± 28.87^#▴^	88.16 ± 15.29	93.42 ± 18.33
BP	59.71 ± 14.39^*▴^	68.05 ± 15.16^#▴^	84.47 ± 21.69	84.00 ± 22.76
GH	45.76 ± 21.52^*▴^	65.61 ± 19.39^#^	70.84 ± 21.17	79.15 ± 16.87
SF	71.95 ± 18.45^*▴^	82.54 ± 15.14^#^	89.25 ± 16.37	90.01 ± 15.42
RE	36.50 ± 42.03^*▴^	79.37 ± 30.69^#▴^	73.68 ± 37.80	91.23 ± 21.78

The scores of PF, RP, BP, and RE in experimental group and the scores of PF, RP, BP, GH, SF, and RE in healthy control group are all non-normal distribution. The * represents P (experimental group–healthy control group) <0.05 before Tai Chi Chuan. The # represents P (after–before) <0.05 in experimental group. The ▴ represents using non-parametric tests based on data distribution.

### The RSNs

**Figure 1:**
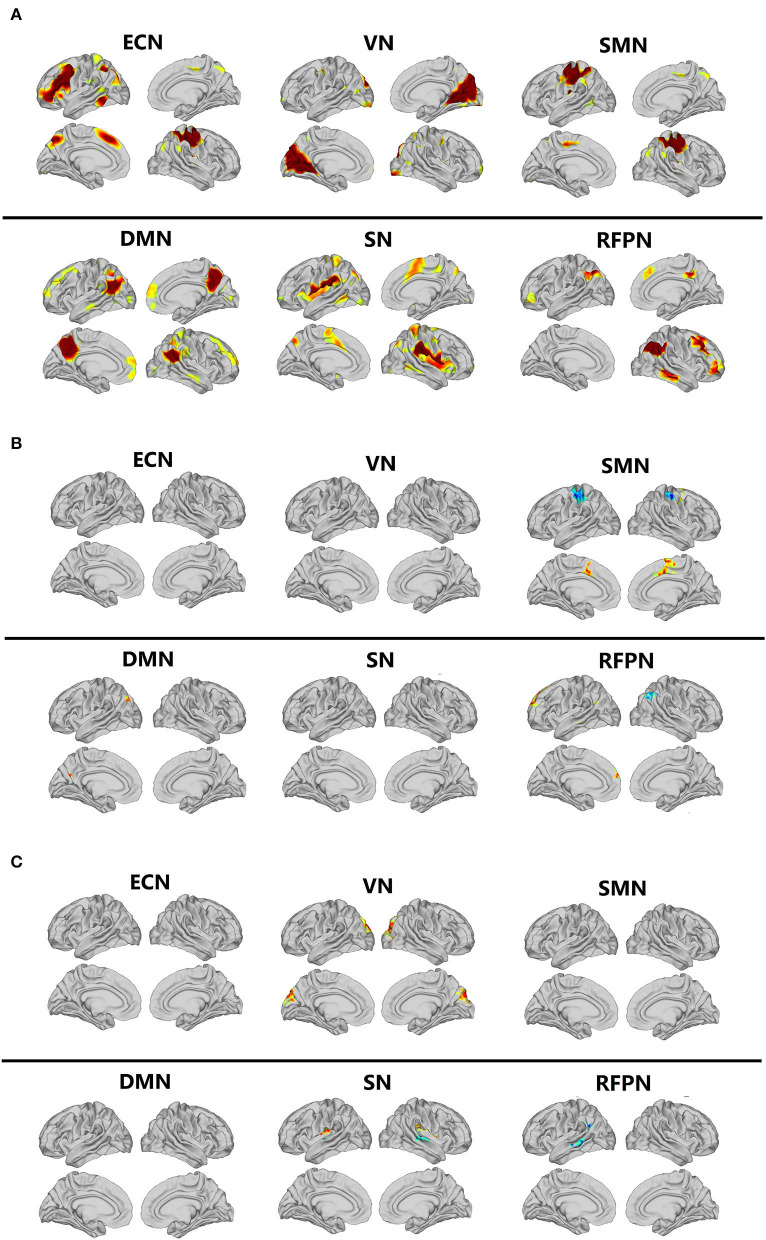
Comparison of resting-state networks. DMN, default mode network; ECN, executive control network; ICA, independent component analysis; RFPN, right frontoparietal network; RSNs, resting-state networks; SMN, sensorimotor network; SN, salience network; VN, visual network. **(A)** The resting-state networks extracted by ICA. **(B)** Comparison of condition effect after and before Tai Chi Chuan in all participants. **(C)** Comparison of interaction effect after and before Tai Chi Chuan in CFS patients and healthy controls.

The magnetic resonance data of all participants were analyzed with independent component analysis (ICA). Six resting brain networks were selected, namely executive control network (ECN), VN, SMN, DMN, SN, and RFPN (Figure 1A). The specific location distribution information of these six networks is shown in the Supplementary Information. For all of the participants, the FC of several brain regions enhanced including the left inferior parietal cortex and posterior cingulate cortex in DMN, the left anterior cingulate/medial prefrontal cortex, inferior parietal cortex, and lateral temporal cortex in RFPN, and the bilateral paracentral lobular/mid-cingulate cortex in SMN and the FC of several brain regions weakened including the right inferior parietal cortex in RFPN and the bilateral somatosensory/motor cortex in SMN after Tai Chi Chuan (Figure 1B and Condition Effect in Table 3). Comparing the enhancement degree of FC in CFS patients with that in healthy controls after and before Tai Chi Chuan, the bilateral posterior opercular cortex in SN and the bilateral cuneus in VN were enhanced better while the left lateral temporal cortex and inferior parietal cortex RFPN and the bilateral superior temporal gyrus in SN were enhanced worse (Figure 1C and Interaction Effect in Table 3).

**Table 3:** The changes of FC in RSNs before and fter Tai Chi Chuan.

**Brain region**	**Side**	**MNI coordinates**			**t value**	**Area (mm^**2**^)**
		**X**	**Y**	**Z**		
**Default mode network–condition effect: After Tai Chi** **>** **Baseline**						
Inferior parietal cortex	L	−9	−65	22	4.58	128
Posterior cingulate cortex	L	−32	−79	38	4.82	120
**Right frontoparietal network–condition effect: After Tai Chi** **>** **Baseline**						
Anterior cingulate/ Medial prefrontal cortex	L	−12	56	24	6.43	831
Inferior parietal cortex	L	−32	−79	38	5.52	182
Lateral temporal cortex	L	−52	−26	−12	5.66	191
**Right frontoparietal network–condition effect: After Tai Chi** **<** **Baseline**						
Inferior parietal cortex	R	46	−52	44	−6.52	613
**Sensorimotor network–condition effect: After Tai Chi** **>** **Baseline**						
Paracentral lobular/ Mid cingulate cortex	L	−10	4	44	7.28	195
Paracentral lobular/ Mid cingulate cortex	R	7	−3	63	8.43	1,210
**Sensorimotor network–condition effect: After Tai Chi** **<** **Baseline**						
Somatosensory/Motor cortex	L	−45	−20	55	−7.12	1,867
Somatosensory/Motor cortex	R	46	−4	50	−6.32	179
**Right frontoparietal network–interaction effect: CFS**_**afterTaiChi**_**-CFS**_**Baseline**_<**HC**_**afterTaiChi**_**-HC**_**Baseline**_						
Lateral temporal cortex	L	−51	−41	−2	−6.26	506
Inferior parietal cortex	L	−48	−54	23	−6.52	244
**Salience network– interaction effect: CFS**_**afterTaiChi**_**-CFS**_**Baseline**_>**HC**_**afterTaiChi**_**-HC**_**Baseline**_						
Posterior opercular cortex	L	−35	−19	20	6.37	427
Posterior opercular cortex	R	42	−1	16	6.37	733
**Salience network– interaction effect: CFS _afterTaiChi_-CFS _Baseline_< HC _afterTaiChi_-HC _Baseline_**						
Superior temporal gyrus	L	−55	−20	0	−5.37	176
Superior temporal gyrus	R	64	−31	11	−7.19	462
**Visual network– interaction effect: CFS _afterTaiChi_-CFS _Baseline_>HC _afterTaiChi_-HC _Baseline_**						
Cuneus	L	−10	−85	33	6.24	742
Cuneus	R	7	−86	31	5.42	984

### GCA Results

GCA indicated that the brain networks of patients with CFS showed different functional connection modes compared with healthy subjects.

**Figure 2:**
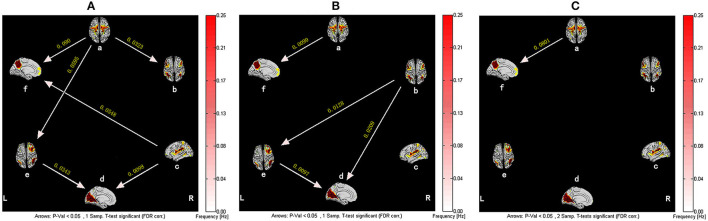
The resting-state effective connectivity comparison between CFS patients and healthy controls. There are six resting-state networks, including: a, SMN; b, ECN; c, SN; d, VN; e, RFPN; f, DMN. Arrow directions represent causal effect between resting-state networks. Yellow numbers indicate strength of causality. Values on the color bar (corresponding with arrow colors) demonstrate frequency at which causality was found. **(A)** One-sample *t*-test result of intergroup intranetwork causal relationship of the healthy controls. **(B)** One-sample *t*-test result of intergroup intranetwork causal relationship of the CFS patients. **(C)** Two-sample *t*-test result of intergroup intranetwork causal relationship of the healthy controls minus the CFS patients.

As shown in Figure 2, in the baseline state, the healthy subjects showed six significant functional relationships, including the causal relationship between SMN and DMN, SMN and RFPN, SMN and ECN, SN and DMN, SN and VN, and RFPN and VN. Among them, SMN and SN were the main output information networks, while DMN and VN were the main input information networks. RFPN was a relay station where information flowed from SMN to VN. On the other hand, there were fewer causal relationships between brain networks of CFS patients, which were mainly manifested in the information exchange among ECN, RFPN, and VN. Meanwhile, the information transmissions of SN to DMN and SN to VN were interrupted, so that SN is isolated from other brain networks. Although the causal relationship between SMN-DMN and RFPN-VN still existed, the frequency of occurrence of them greatly decreased. Compared with healthy subjects, the causal relationship between SMN and DMN in CFS patients was significantly different (*P* < 0.05, False Discovery Rate Correction), which was mainly manifested in the weakening of the effective connection between SMN and DMN.

**Figure 3:**
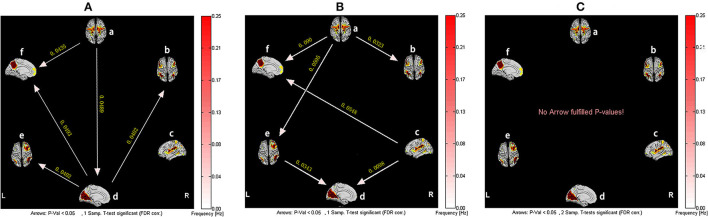
The resting-state effective connectivity comparison of healthy subjects before and after Tai Chi Chuan training. There are six resting-state networks, including: a, SMN; b, ECN; c, SN; d, VN; e, RFPN; f, DMN. Arrow directions represent causal effect between resting-state networks. Yellow numbers indicate strength of causality. Values on the color bar (corresponding with arrow colors) demonstrate frequency at which causality was found. **(A)** One-sample *t*-test result of intergroup intranetwork causal relationship of the healthy controls after Tai Chi Chuan training. **(B)** One-sample *t*-test result of intergroup intranetwork causal relationship of the healthy controls before Tai Chi Chuan training. **(C)** Paired *t*-test result of intragroup intranetwork causal relationship of after Tai Chi Chuan training minus before Tai Chi Chuan training for healthy controls.

For healthy subjects, after 1 month of Tai Chi Chuan training, VN became the network with the most output information. The information transmission modes in the baseline state of SMN-ECN and SMN-RFPN were changed to SMN-VN-ECN and SMN-VN-RFPN, both used VN as a relay station. Interestingly, the causal relationship between RFPN and VN was reversed compared to the baseline state. However, before and after Tai Chi Chuan training, healthy subjects did not show such a significant difference in the causal relationship of brain networks (Figure 3).

**Figure 4:**
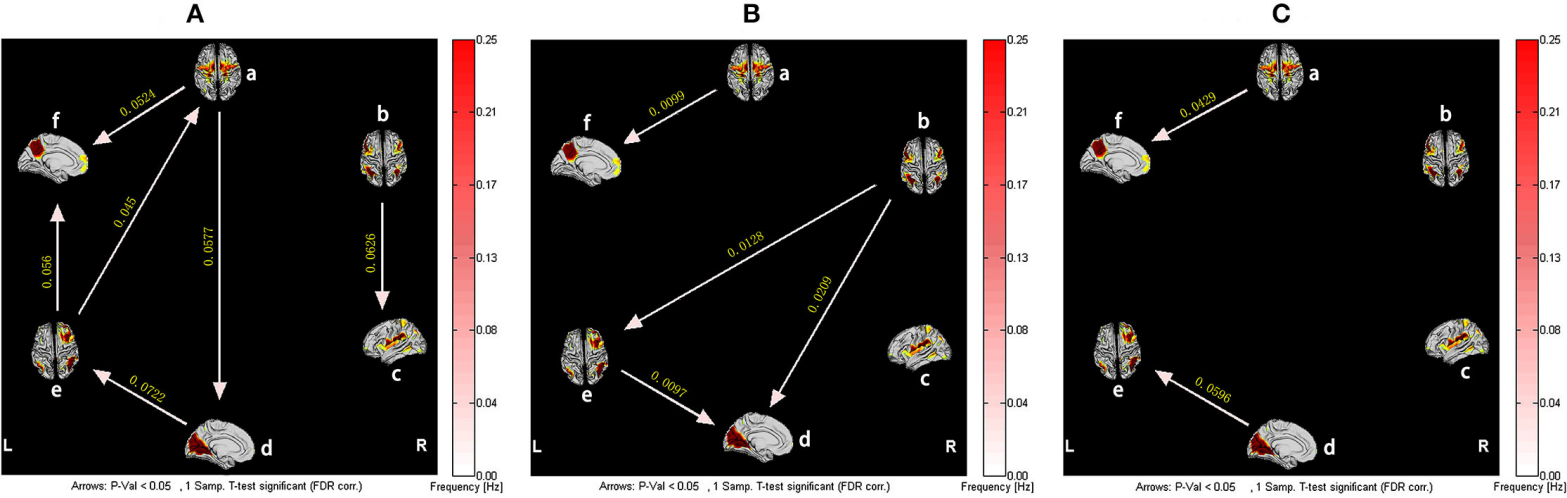
The resting-state effective connectivity comparison of CFS patients before and after Tai Chi Chuan training. There are six resting-state networks, including: a, SMN; b, ECN; c, SN; d, VN; e, RFPN; f, DMN. Arrow directions represent causal effect between resting-state networks. Yellow numbers indicate strength of causality. Values on the color bar (corresponding with arrow colors) demonstrate frequency at which causality was found. **(A)** One-sample *t*-test result of intergroup intranetwork causal relationship of the CFS patients after Tai Chi Chuan training. **(B)** One-sample *t*-test result of intergroup intranetwork causal relationship of the CFS patients before Tai Chi Chuan training. **(C)** Paired *t*-test result of intragroup intranetwork causal relationship of after Tai Chi Chuan training minus before Tai Chi Chuan training for CFS patients.

However, for the patients with CFS, after training in Tai Chi Chuan, the information flow between brain networks was more abundant. Among them, SMN and RFPN became core networks outputting the most information, while DMN received the most information. In addition, it is not difficult to find the causal relationships among SMN-VN-RFPN, which realized the circulation of information. Although the connections between ECN-RFPN and ECN-VN were interrupted, the causality between SN and ECN is established. It is worth noting that, similar to healthy subjects, CFS patients also experienced the reversal of the causal relationship between RFPN and VN after training. Compared with before training, the effective connections of CFS patients from SMN to DMN and VN to RFPN were significantly enhanced after training (*P* < 0.05, False Discovery Rate Correction) (Figure 4).

### Correlations

**Figure 5:**
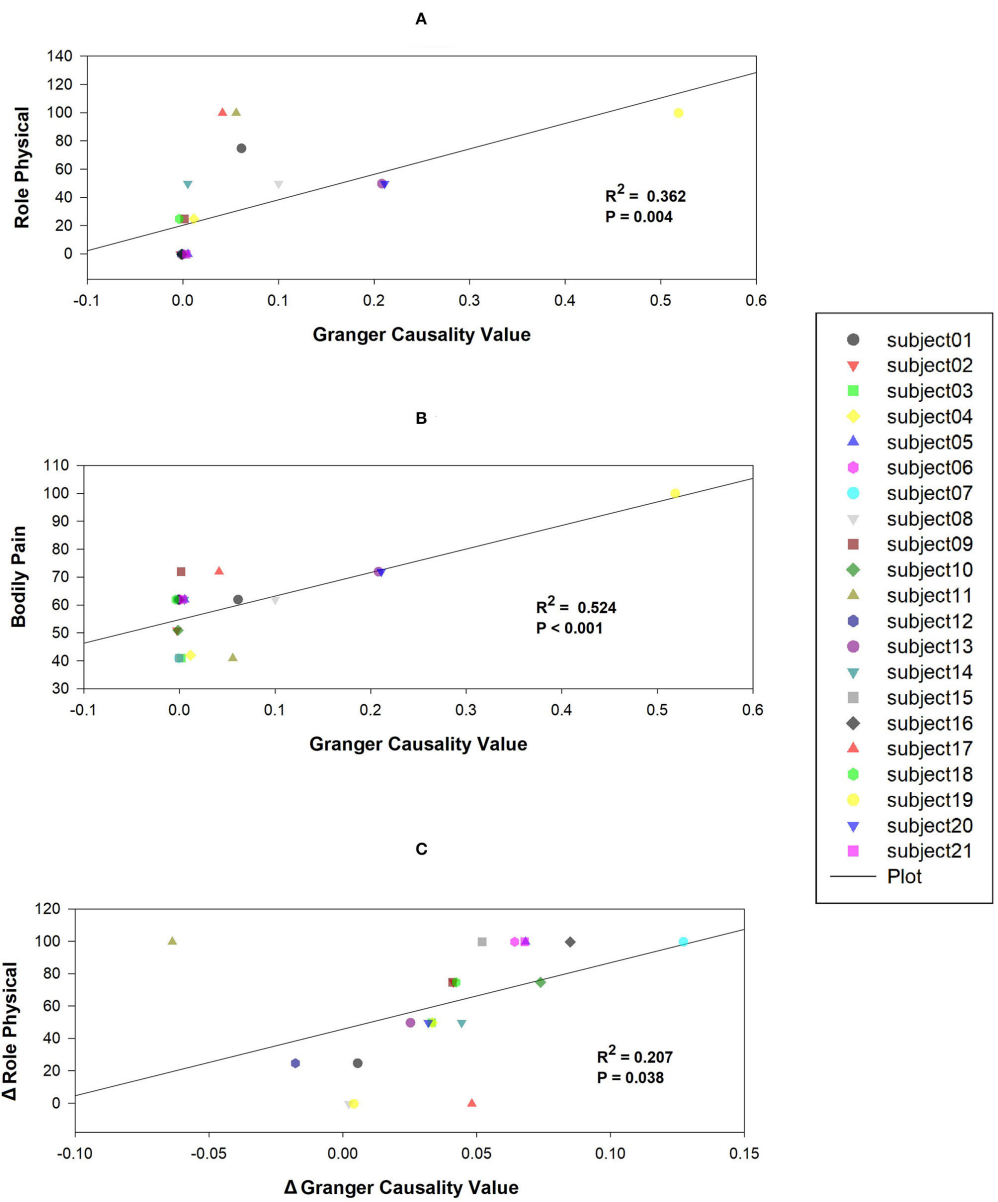
Correlations between mean Granger causality values within abnormal casual connectivity and the scores of Role Physical and Bodily Pain. **(A)** The mean Granger causality values from SMN to DMN were positively associated with the score of Role Physical (*R*^2^= 0.362, *P* = 0.004). **(B)** The mean Granger causality values from SMN to DMN were positively associated with the score of Bodily Pain (*R*^2^= 0.524, *P* < 0.001). **(C)** The mean Granger causality difference from SMN to DMN was positively associated with the score of Role Physical difference (*R*^2^= 0.207, *P* = 0.038).

The results showed that the mean causal value of SMN-DMN was positively correlated with the scores of RP and BP (Figures 5A,B). Besides, the correlation analysis between SMN-DMN Granger causality value difference and RP difference before and after training showed that there was a significant positive correlation between them (Figure 5C).

## Discussion

Tai Chi Chuan was introduced as an exercise therapy, which has been proved to have the ability to promote brain plasticity (45). Accordingly, to explore the central mechanism of Tai Chi Chuan in improving CFS, its role in promoting the plasticity of CFS patients' brain function networks was investigated for the first time in our study. Our data indicated that, compared with healthy people, patients with CFS had lower HRQoL and weaker Granger causality between RSNs. Tai Chi Chuan training could just adjust these differences, especially the information flow between SMN and DMN.

In our results, the SF-36 scale score of patients was significantly lower than that of healthy controls, which is consistent with the previous results (46, 47), indicating that the HRQoL of CFS patients has been seriously affected. However, after training in Tai Chi Chuan, the SF-36 score of CFS patients had been significantly improved compared with the baseline state, which supports the notion that Tai Chi Chuan has an obvious effect in improving HRQoL of CFS patients.

CFS, as a complex disorder composed of various symptoms, is related to many brain functions such as sensory, motor, and cognition. Our results showed a change in the effective connection between SMN and DMN in CFS patients compared with the healthy control group, which was in line with previous studies (14–16). The anterior central gyrus, posterior central gyrus, and auxiliary motor areas are the core areas of SMN, which are related to somatosensory and motor functions. Previous structural and functional MRI studies have illustrated the abnormalities of SMN. For instance, others have verified the increase in thickness of anterior central gyrus cortex in CFS patients (48). It was also established that the FC between the left anterior middle cingulate gyrus and SMN decreased in CFS patients (16). Another fMRI study (49) based on arterial spin labeling (ASL) showed that the FC between the parahippocampal gyrus, right anterior central gyrus, and the left posterior central gyrus in CFS patients was weakened. DMN plays an indispensable role in the study of resting brain networks. It is activated when the brain does not receive any stimulation or tasks, but weakened when it receives stimulation or tasks. Also, the degree of negative activation of DMN increases with the increase of task difficulty (50). DMN plays a vital role in spontaneous introspection (51) as well as advanced cognitive function (52). From the aspect of brain structures, anterior cuneiform lobe and PCC are important areas, which are related to information transmission and sensory information integration (53). The irregular activity of PCC in CFS patients observed in the past (14) may be an important reason for influencing the information transmission in DMN. This abnormal information transmission between SMN and DMN leads to the obstacle of integration of body movement and sensory information, and finally gives rise to two obvious manifestations of CFS, namely body pain and exercise intolerance. Apart from this, the correlation analysis also showed that the causal relationship between SMN and DMN was positively correlated with the RP and BP scores of SF-36 scale. Thus, the stronger the effective connection between the two networks, the stronger the physiological and somatosensory functions of patients. Hence, we suggest that the reduction of information integration between DMN and SMN may be the core mechanism of CFS.

Gratifyingly, after a month of training in Tai Chi Chuan, the SF-36 scale reflected the improvement of HRQoL of CFS patients, and the information transmission between SMN and DMN was stronger than before. Correlation analysis also showed that the change of effective connection strength between SMN and DMN had a positive correlation with the change in physiological function. A similar effect of Tai Chi Chuan has been described in another neuroimaging study (54) in which the FC between DMN and SMN was weak before rehabilitation therapy for stroke patients but enhanced after rehabilitation therapy, and the interaction between the two networks was positively correlated with motor function. In our results, it was the FC of the left inferior parietal cortex and PCC in DMN that was enhanced after Tai Chi Chuan. Therein, the inferior parietal cortex makes a critical contribution to spatial processing and cognition especially attention processing (55, 56), and PCC has a pivotal role in conscious awareness and is inactivated with painful stimulation (57). Other scholars also confirmed that Tai Chi Chuan was conducive to the functional consistency in the posterior central gyrus belonging to the SMN of employees (58), which is different from ours. What we have found is the enhanced FC of the bilateral paracentral lobular/mid-cingulate cortex and the weakened FC of the bilateral somatosensory/motor cortex in SMN. Therein, the mid-cingulate cortex is considered to be helpful to exert attentional control (59), and the interaction between somatosensory and motor cortex realized sensory processing and movement control (60, 61). These dissimilarities may own to the difference of the participants included. Taken together, Tai Chi Chuan promoted the plasticity of brain function by regulating the causal relationship between brain networks. Since there are pretty rare studies about the effect of Tai Chi Chuan in CFS, our results need to be verified by more research.

Remarkably, the reversed and strengthened causal relationship between RFPN and VN in CFS patients who often suffer from cognitive disorders (62–66) was also uncovered in our observations. In like manner, another longitudinal study (21) also found that Tai Chi Chuan changed the FC of the dorsolateral prefrontal cortex, which is a key area of both the cognitive control network and frontoparietal network (FPN). Our results, by contrast, showed the enhanced FC of the left anterior cingulate/medial prefrontal cortex, inferior parietal cortex, and lateral temporal cortex and the weakened FC of the right inferior parietal cortex in RFPN. These dissimilarities may be caused by the participants we included being CFS patients and healthy people, rather than the elderly. The information transmission between FPN and VN is closely related to cognitive function, especially the attention to visual space (67, 68). Among them, FPN plays a vital role in spatial attention and motion control, such as target-oriented hand and eye movement (69), which are exactly what the Tai Chi Chuan exercise requires. What's more, as shown in Table 3, enhancing the FC of the bilateral cuneus in VN by Tai Chi Chuan was more effective in CFS patients, related to visual information processing and visuomotor planning (70). Consequently, we speculate that the change in the causal relationship between RFPN and VN is the neural basis for Tai Chi Chuan to improve the cognitive function of CFS patients, especially visual-spatial attention.

There are still a few inconsistencies compared with previous studies (71–73). A prior study revealed that the FC of DMN is enhanced in female CFS patients (71). Whereas, further study (73) did not show any significant FC change of DMN in CFS adolescent patients compared with healthy controls. Differently, only significant FC change of SN in adolescent patients was found in another study (72). These inconsistencies may be due to different subjects and diagnostic criteria. Studies have proved that teenagers' brain networks are constantly changing (74), and a large-scale cross-sectional study (75) has also proved that there are differences in functional connections between male and female brain networks. Here, we mainly applied male and female gender in the age of 25 to 65 years, which would raise a significant difference to the only-female group. Moreover, different diagnostic standards, heterogeneity of population, and brain function analyses unavoidably contribute to the differences in these studies.

There are also several limitations in our research. First, the sample size is relatively small. Only 21 patients and 19 healthy people were included in our study. Second, the changes in RSNs related to cognitive function were found, but the indicators related to cognitive function were lacking. Third, GCA may not distinguish the direct causality caused by the action of the intermediate network, introducing a limited result in the brain analysis. Fourth, performing a double-blind trial on physiotherapy interventions is impossible, so there might be placebo effects. Last but not least, there's no similar large sample study before for us to refer to, and the results need to be repeated. Future research should increase the sample size, improve the analysis method, supplement the correlation analysis between the changes in brain networks and the improvement of cognition function, and compare the effect of conventional exercise therapy with Tai Chi Chuan.

## Conclusion

In conclusion, we recruited patients with CFS and healthy controls for fMRI scanning before and after 1-month-long training in Tai Chi Chuan. ICA and GCA were used to extract the RSNs and the changes of FC in these networks were also analyzed. Compared to the healthy control group, the causal relationship between SMN and DMN was weakened, which could be improved by Tai Chi Chuan. The findings suggest that the change of Granger causality between SMN and DMN may be a readout parameter of CFS. Tai Chi Chuan may promote the functional plasticity of brain networks in patients with CFS by regulating the information transmission between RSNs.

## Data Availability Statement

The original contributions presented in the study are included in the article/Supplementary Material, further inquiries can be directed to the corresponding authors.

## Ethics Statement

The study involving human participants was reviewed and approved by Medical Ethics Committee of Dongzhimen Hospital of Beijing University of Chinese Medicine. The patients/participants provided their written informed consent to participate in this study.

## Author Contributions

KL and YZ: conceptualization, methodology, and formal analysis. YL, KW, XH, and TX: data collection and research performance. YL and KW: writing the original draft. KL: revising the original draft. YZ and ZL: research supervision. All authors contributed to the article and approved the submitted version.

## Funding

This study was supported by the National Natural Science Foundation (Grant No. 82004437) and the Beijing Natural Science Foundation (Grant No. 7204277).

## Conflict of Interest

The authors declare that the research was conducted in the absence of any commercial or financial relationships that could be construed as a potential conflict of interest.

## Publisher's Note

All claims expressed in this article are solely those of the authors and do not necessarily represent those of their affiliated organizations, or those of the publisher, the editors and the reviewers. Any product that may be evaluated in this article, or claim that may be made by its manufacturer, is not guaranteed or endorsed by the publisher.
