# Supplementary material for: Altered Effective Connectivity of Resting-State Networks by Tai Chi Chuan in Chronic Fatigue Syndrome Patients: A Multivariate Granger Causality Study
Source: Front Neurol. 2022 Jun 3;13:858833. doi: 10.3389/fneur.2022.858833 (PMC9203735; doi:10.3389/fneur.2022.858833)
Supplement: Supplementary file 1 [file Table_1.DOCX]

Supplementary Material

## Supplementary Tables

**Table 1 Spatial positional distributions of brain networks**

| Cluster Number | Regin | Herm | MNI coordinates | | | t value | Area  (mm^2^) |
| --- | --- | --- | --- | --- | --- | --- | --- |
|  |  |  |  |  |  |  |  |
|  |  |  | **X** | **Y** | **Z** |  |  |
| ECN | | | | | | | |
| 1 | Frontal Cortex | L | 41 | 26 | 234 | 11.16 | 4199 |
| 2 | IntraParietal Cortex | L | 33 | 65 | 41 | 16.38 | 2003 |
| 3 | Temporal Cortex | L | 54 | 51 | 8 | 5.22 | 612 |
| 4 | Anterior Cingulate | L | 7 | 26 | 47 | 6.45 | 550 |
| 5 | Anterior Ventral Insular | L | 28 | 22 | 1 | 4.23 | 95 |
| 6 | Frontal Cortex | R | -46 | -25 | -28 | 10.05 | 2081 |
| 7 | IntraParietal Cortex | R | -35 | -66 | -43 | 10.01 | 611 |
| 8 | Anterior cingulate | R | -7 | -40 | -40 | 5.569 | 460 |
| 9 | Anterior Ventral Insular | R | -30 | -22 | -1 | 4.5 | 157 |
| VN | | | | | | | |
| 1 | Occipital Cortex (Cuneus) | L | -13 | -73 | 12 | 15.6 | 9401 |
| 2 | Occipital Cortex (Cuneus) | R | 20 | -65 | 7 | 19.58 | 6823 |
| SMN | | | | | | | |
| 1 | Precentral Cortex /Postcentral Cortex | L | -44 | -25 | 60 | 26.94 | 4550 |
| 2 | Precentral Cortex / Postcentral Cortex | R | 45 | -21 | 61 | 22.15 | 4105 |
| RFPN | | | | | | | |
| 1 | Inferior Parietal Cortex/ Superior Parietal Cortex | L | -42 | -64 | 45 | 8.1 | 1029 |
| 2 | Prefrontal Cortex | R | 35 | 18 | 49 | 10.27 | 3667 |
| 3 | Inferior Parietal Cortex/ Superior Parietal Cortex | R | 48 | -58 | 44 | 19.45 | 2159 |
| 4 | Superior Temporal Cortex | R | 64 | -32 | -8 | 8.05 | 1244 |
| DMN | | | | | | | |
| 1 | Inferior Parietal Cortex/ Superior Parietal Cortex | L | -52 | -54 | 21 | 12.62 | 3152 |
| 2 | Posterior Cingulate Cortex | L | -8 | -61 | 42 | 16.05 | 1930 |
| 3 | Anterior Cingulate Cortex | L | -26 | 27 | 34 | 3.44 | 2260 |
| 4 | Inferior Parietal Cortex/ Superior Parietal Cortex | R | 50 | -49 | 24 | 13.64 | 1665 |
| 5 | Posterior Cingulate Cortex | R | 5 | -61 | 37 | 16.53 | 1350 |
| 6 | Anterior Cingulate Cortex | R | 25 | 26 | 37 | 3.92 | 1738 |
| 7 | Superiror Temporal Cortex | R | 60 | -16 | -14 | 3.3 | 775 |
| SN | | | | | | | |
| 1 | Insular | L | -62 | -30 | 21 | 10.83 | 5207 |
| 2 | Middle Cingulate Cortex | L | -10 | 7 | 40 | 3.29 | 867 |
| 3 | Precentral Cortex/Postcentral Cortex | L | -32 | -73 | 43 | 4.53 | 527 |
| 4 | Insular | R | -60 | -29 | 20 | 10.8 | 6237 |
| 5 | Precentral Cortex/Postcentral Cortex | R | 24 | -46 | 62 | 4.4 | 1312 |
| 6 | Middle Cingulate Cortex | R | 7 | 3 | 54 | 3.49 | 1235 |

ECN, executive-control network; VN, visual network; SMN, sensorimotor network; RFPN, right frontoparietal network; DMN, default mode network; SN, salience network.
